# Drug sensitivity prediction with high-dimensional mixture regression

**DOI:** 10.1371/journal.pone.0212108

**Published:** 2019-02-27

**Authors:** Qianyun Li, Runmin Shi, Faming Liang

**Affiliations:** 1 Department of Biostatistics, University of Florida, Gainesville, FL 32611, United States of America; 2 Department of Statistics, University of Florida, Gainesville, FL 32611, United States of America; 3 Department of Statistics, Purdue University, West Lafayette, IN 47906, United States of America; New Jersey Institute of Technology, UNITED STATES

## Abstract

This paper proposes a mixture regression model-based method for drug sensitivity prediction. The proposed method explicitly addresses two fundamental issues in drug sensitivity prediction, namely, population heterogeneity and feature selection pertaining to each of the subpopulations. The mixture regression model is estimated using the imputation-conditional consistency algorithm, and the resulting estimator is consistent. This paper also proposes an average-BIC criterion for determining the number of components for the mixture regression model. The proposed method is applied to the CCLE dataset, and the numerical results indicate that the proposed method can make a drastic improvement over the existing ones, such as random forest, support vector regression, and regularized linear regression, in both drug sensitivity prediction and feature selection. The *p*-values for the comparisons in drug sensitivity prediction can reach the order *O*(10^−8^) or lower for the drugs with heterogeneous populations.

## Introduction

Drug sensitivity prediction is an integral part of precision medicine which, unlike the traditional one-size-fits-all approach, tailors therapy to each patient by accounting for their heterogeneity in e.g. clinic, genomic, and environments. During the past decade, the study of drug sensitivity prediction has received a boost due to the ever-growing interest in precision medicine and the availability of large-scale pharmacogenomics datasets. Various drug sensitivity prediction methods have been proposed in the literature, including regularized linear regression, support vector regression, and random forest, among others. Refer to [1] for an overview of these methods.

The regularized linear regression is to model the effect of each feature in a linear function. In this paper, we refer to features as the patient attributes under investigation, e.g., age, gender, genes, SNPs, copy number variants, or some demographic variables. Since, in the current pharmacogenomics datasets, the number of genomic features is often much larger than the number of samples (a.k.a. small-*n*-large-*p*), the regression model is ill-posed and a regularization term has to be included to enable its solution. Examples of regularized linear regression used in drug sensitivity prediction include ridge regression [2] and elastic net [3, 4], which employ a *l*_2_-penalty and a combination of *l*_1_- and *l*_2_-penalties, respectively. A systematic study for the two models in drug sensitivity prediction has been reported in [5]. Other regularized linear regression methods, such as Lasso [6], SCAD [7], MCP [8] and rLasso [9], can also be applied to this problem.

Support vector regression is a kernel-based method, which can model via kernels the effect of each feature in a nonlinear function. The commonly used kernels include the linear kernel, polynomial kernel, radial basis kernel, and sigmoidal kernel. Support vector regression has been used for drug sensitivity prediction in [5, 10–12].

The random forest models the data by a set of regression trees, where the training set for each tree is selected using bootstrap sampling from the original sample set, and the features considered for partitioning at each node are a random subset of the original set of features. The random forest falls into the class of nonlinear regression models, where the samples are partitioned at each node of the binary tree based on the value of one selected feature. It tends to have high accuracy prediction and can handle a large number of features due to the embedded feature selection in the model generation process. The random forest model can also be viewed as a mixture model, with each tree corresponding to a submodel. The random forest is one of the top performing algorithms in the NCI-DREAM drug sensitivity prediction challenge [11, 13] and has been used in multiple other drug sensitivity studies [12, 14–16].

Although these methods can work reasonably well for some datasets, none of them has directly addressed the issue of population heterogeneity, that is, different patients may have different disease-causing factors. It is known that the population heterogeneity forms the biological basis of precision medicine. Under population heterogeneity, selection of the features that affect the drug sensitivity has not been addressed either. This article aims to address the two issues simultaneously. We propose to model the drug sensitivity using a high-dimensional mixture linear regression, which directly addresses the issue of population heterogeneity. When estimating the mixture model, the samples will be clustered into different groups and different drug sensitive features will then be selected for each group. Although the random forest can be viewed as a mixture model and has an embedded feature selection procedure, it does not explicitly group the samples and select different features for different groups. We demonstrate the performance of the proposed method through simulation and analysis of the CCLE dataset. The numerical results indicate that the proposed method can make a drastic improvement over the existing ones, including random forest, support vector regression and regularized linear regression, in drug sensitivity prediction.

## Materials and methods

### High-dimensional mixture regression

Suppose that we have collected a set of random samples (***x***_1_, *y*_1_), …, (***x**_n_*, *y_n_*), where $y_{i}\in R$ and $x_{i}\in R^{p}$ for *i* = 1, ⋯, *n*, and *n* is the sample size. Each *y*_*i*_ is independently drawn from a finite Gaussian mixture distribution with the density function given by

$$\begin{array}{c} f\left(y_{i}|\theta \right)=\sum_{k=1}^{K}\pi_{k}\phi \left(y_{i}|x_{i}\beta_{k},\sigma_{k}^{2}\right), \end{array}$$ (1)

where ***θ*** = (***β***_1_, …, ***β**_K_*; *σ*_1_, …, *σ_K_*; *π*_1_, …, *π*_*K*−1_) denotes the parameter vector, *ϕ*(⋅|*μ*, *σ*^2^) is the Gaussian density function with mean *μ* and variance *σ*^2^, *π*_*k*_ is the mixing proportion, and ***β**_k_* is a (*p* + 1)-dimensional vector whose first component corresponds to the intercept term and others correspond to the regression coefficients of the *p* features. Further, we assume that *p* can be much greater than *n* and it can grows with *n* in a polynomial rate *O*(*n*^*γ*^) for some constant *γ* > 0. To indicate the dependence of *p* on *n*, we may rewrite *p* as *p*_*n*_ in the remaining part of this paper. In addition, we assume that ***β**_k_* = (*β*_*k*0_, *β*_*k*1_, …, *β_kp_*) is sparse for each *k*, i.e.,

$$\begin{array}{c} \sum_{i=1}^{p_{n}}I\left(\beta_{ki}\neq 0\right)<\infty ,\;\text{as}\;n\to \infty . \end{array}$$

Our goal is to cluster the *n* samples into *K* groups, with each corresponding to a subpopulation in (1), and identify the nonzero components of ***β**_k_* for each *k*.

For the low-dimensional problems for which *p*_*n*_ is much smaller than *n* or, more precisely, the dimension of ***θ*** is smaller than *n*, the mixture regression model can be estimated using the EM algorithm [17] by treating the cluster membership of each sample as missing data. The EM algorithm leads to an maximum likelihood estimate (MLE) of ***θ***.

When the dimension of ***θ*** is greater than *n*, the EM algorithm cannot be used any more, as the problem is ill-posed and the MLE might no longer to consistent to the true parameter. To address this issue, certain type of regularization has to be imposed on ***θ***. For example, [18] proposed to estimate ***θ*** by maximizing a penalized likelihood function, which is to set

$$\begin{array}{c} \hat{\theta }=\text{arg}\;\underset{\theta }{\text{max}}\{\sum_{i=1}^{n}\text{log}\left\{\sum_{k=1}^{K}\pi_{k}\phi \left(y_{i}|x_{i}\beta_{k},\sigma_{k}^{2}\right)\right\}-P_{\lambda }\left(\theta \right)\}, \end{array}$$ (2)

where *P*_λ_(***θ***) is the penalty function and λ is the regularization parameter. The algorithm has been implemented in the R package *fmrs* [19], where different penalty functions have been considered, including the Lasso penalty [6], adaptive Lasso penalty [20], MCP penalty [8], SCAD penalty [7], and the hard penalty [18]. Although the method can be shown to produce a consistent estimate of ***θ*** under appropriate conditions, its convergence rate seems low. That is, it needs a large sample size to produce a good estimate of ***θ***. In the next subsection, we propose a new method to estimate the mixture regression, which, as indicated by our numerical examples, significantly outperforms the penalized likelihood method.

### Imputation-conditional consistency algorithm

The imputation-conditional consistency (ICC) algorithm [21] is a general algorithm for dealing with high-dimensional missing data problems. Let ***X***^obs^ denote the observed data, and let ***X***^mis^ denote the missed data. Suppose that ***θ*** has been partitioned into *b* blocks ***θ*** = (***θ***^(1)^, …, ***θ***^(*b*)^). Let $\theta_{n}^{\left(t\right)}=\left(\theta_{n}^{\left(t,1\right)},\ldots ,\theta_{n}^{\left(t,b\right)}\right)$ denote the estimate of ***θ*** obtained at iteration *t*, where the subscript *n* indicates its dependence on the samples. The imputation-conditional consistency (ICC) algorithm works by iterating between the following steps:

- **I-step**. Draw ${\tilde{x}}^{\text{mis}}$ from the predictive distribution $h(x^{\text{mis}}|x^{\text{obs}},\theta_{n}^{\left(t,1\right)},\ldots ,\theta_{n}^{\left(t,b\right)})$ given ***x***^obs^ and the current estimate $\theta_{n}^{\left(t\right)}=\left(\theta_{n}^{\left(t,1\right)},\ldots ,\theta_{n}^{\left(t,b\right)}\right)$.
- **CC-step**. Based on the pseudo-complete data $\tilde{x}=\left(x^{\text{obs}},{\tilde{x}}^{\text{mis}}\right)$, do the following:
  (1) Conditioned on $\left(\theta_{n}^{\left(t,2\right)},\ldots ,\theta_{n}^{\left(t,b\right)}\right)$, find $\theta_{n}^{\left(t+1,1\right)}$ which forms a consistent estimate of
    $$\begin{array}{c} \theta_{*}^{\left(t,1\right)}=\text{arg}\;\underset{\theta_{n}^{{\left(t,1\right)}^{\prime }}}{\text{max}}E_{\theta_{n}^{{\left(t,1\right)}^{\prime }},\ldots ,\theta_{n}^{\left(t,b\right)}}\;\text{log}\;f\left(\tilde{x}|\theta_{n}^{{\left(t,1\right)}^{\prime }},\theta_{n}^{\left(t,2\right)},\ldots ,\theta_{n}^{\left(t,b\right)}\right), \end{array}$$
    where the expectation is taken with respect to the joint distribution of $\tilde{x}=\left(x^{\text{obs}},{\tilde{x}}^{\text{mis}}\right)$ and the subscript of *E* gives the current estimate of ***θ***.
  (2) Conditioned on $\left(\theta_{n}^{\left(t+1,1\right)},\theta_{n}^{\left(t,3\right)},\ldots ,\theta_{n}^{\left(t,b\right)}\right)$, find $\theta_{n}^{\left(t+1,2\right)}$ which forms a consistent estimate of
    $$\begin{array}{c} \theta_{*}^{\left(t,2\right)}=\text{arg}\;{\text{max}}_{\theta_{n}^{{\left(t,2\right)}^{\prime }}}E_{\theta_{n}^{\left(t+1,1\right)},\theta_{n}^{{\left(t,2\right)}^{\prime }},\theta_{n}^{\left(t,3\right)},\ldots ,\theta_{n}^{\left(t,b\right)}}\;\text{log}\;f\left(\tilde{x}|\theta_{n}^{\left(t+1,1\right)},\theta_{n}^{{\left(t,2\right)}^{\prime }},\theta_{n}^{\left(t,3\right)},\ldots ,\theta_{n}^{\left(t,b\right)}\right). \end{array}$$……
  (b) Conditioned on $\left(\theta_{n}^{\left(t+1,1\right)},\ldots ,\theta_{n}^{\left(t+1,b-1\right)}\right)$, find $\theta_{n}^{\left(t+1,b\right)}$ which forms a consistent estimate of
    $$\begin{array}{c} \theta_{*}^{\left(t,b\right)}=\text{arg}\;{\text{max}}_{\theta_{n}^{{\left(t,b\right)}^{\prime }}}E_{\theta_{n}^{\left(t+1,1\right)},\ldots ,\theta_{n}^{\left(t+1,b-1\right)},\theta_{n}^{{\left(t,b\right)}^{\prime }}}\;\text{log}\;f\left(\tilde{x}|\theta_{n}^{\left(t+1,1\right)},\ldots ,\theta_{n}^{\left(t+1,b-1\right)},\theta_{n}^{{\left(t,b\right)}^{\prime }}\right). \end{array}$$

As indicated by the algorithm, to find a consistent estimate of $\theta_{*}^{\left(t,i\right)}$, the ideal objective function is

$$\begin{array}{c} E_{\theta_{n}^{\left(t+1,1\right)},\ldots ,\theta_{n}^{\left(t+1,i-1\right)},\theta_{n}^{{\left(t,i\right)}^{\prime }},\theta_{n}^{\left(t,i+1\right)},\ldots ,\theta_{n}^{\left(t,b\right)}}\;\text{log}\;f\left(\tilde{x}|\theta_{n}^{\left(t+1,1\right)},\ldots ,\theta_{n}^{\left(t+1,i-1\right)},\theta_{n}^{{\left(t,i\right)}^{\prime }},\theta_{n}^{\left(t,i+1\right)},\ldots ,\theta_{n}^{\left(t,b\right)}\right), \end{array}$$

but which cannot be directly evaluated. Practically, the consistent estimate of each block can be obtained by maximizing a regularized conditional likelihood function, i.e., setting the estimate

$$\begin{array}{c} \begin{array}{cc} \theta_{n,p}^{\left(t+1,i\right)} & =\text{arg}\;\underset{\theta_{n}^{{\left(t,i\right)}^{\prime }}}{\text{max}}\{\frac{1}{n}\sum_{j=1}^{n}\;\text{log}\;f\left(x_{j}^{\text{obs}},{\tilde{x}}_{j}^{\text{mis}}|\theta_{n}^{\left(t+1,1\right)},\ldots ,\theta_{n}^{\left(t+1,i-1\right)},\theta_{n}^{{\left(t,i\right)}^{\prime }},\theta_{n}^{\left(t,i+1\right)},\ldots ,\theta_{n}^{\left(t,b\right)}\right)\\ & -P_{\lambda_{i}}\left(\theta^{{\left(t,i\right)}^{\prime }}\right)\}, \end{array} \end{array}$$ (3)

where $P_{\lambda_{i}}\left(\cdot \right)$ denotes the regularization/penalty function used for block *i*. Let $\left\{{\tilde{x}}_{t}^{\text{mis}}:t=1,2,\ldots \right\}$ denote the sequence of imputed data during the iterations. Similar to the stochastic EM algorithm [22, 23], it is easy to see that the sequences, $\left\{{\tilde{x}}_{t}^{\text{mis}}:t=1,2,\ldots \right\}$ and $\left\{\theta_{n}^{\left(t\right)}:t=1,2,\ldots \right\}$, form two interleaved Markov chains:

$$\begin{array}{c} \theta_{n}^{\left(1\right)}\to {\tilde{x}}_{1}^{\text{mis}}\to \theta_{n}^{\left(2\right)}\to {\tilde{x}}_{2}^{\text{mis}}\to \cdots \cdots \to \theta_{n}^{\left(n\right)}\to {\tilde{x}}_{n}^{\text{mis}}\to \cdots \cdots . \end{array}$$

The convergence of these two Markov chains has been rigorously studied in [21] under quite general conditions. Theorem 5 and Theorem 6 of [21] show that the Markov chain $\left\{\theta_{n}^{\left(t\right)}:t=1,2,\ldots \right\}$ has a stationary distribution and the mean of the stationary distribution forms a consistent estimate of the true parameter θ*.

For the mixture regression model, if we treat the cluster membership of each sample as missing data, then the ICC algorithm can be applied. Let *τ*_1_, …, *τ*_*n*_ denote the cluster membership variable of the *n* samples. Then

$$\begin{array}{c} P\left(\tau_{i}=k|\theta \right)=\frac{\pi_{k}\phi \left(y_{i}|x_{i}\beta_{k},\sigma_{k}^{2}\right)}{\sum_{j=1}^{K}\pi_{j}\phi \left(y_{i}|x_{i}\beta_{j},\sigma_{j}^{2}\right)},\;k=1,2,\ldots ,K, \end{array}$$ (4)

for *i* = 1, 2, …, *n*. Let $p_{i}^{\left(t\right)}=\left\{P\left(\tau_{i}=1|\theta^{\left(t\right)}\right),\ldots ,P\left(\tau_{i}=K|\theta^{\left(t\right)}\right)\right\}$, and let $\tau_{i}^{\left(t\right)}$ denote the cluster membership imputed for sample *i* at iteration *t*. Applying the ICC algorithm to the mixture regression model leads to the following procedure:

- (I-step) Simulate $\tau_{i}^{\left(t+1\right)}∼\text{Multinomial}\left(1,p_{i}^{\left(t\right)}\right)$ for *i* = 1, 2, …, *n*. Define the subsets $\chi_{k}^{\left(t+1\right)}=\left\{i\in \left\{1,2,\ldots ,n\right\}:\tau_{i}^{\left(t+1\right)}=k\right\}$ for *k* = 1, 2, …, *K*.
- (CC-step) For each component *k* = 1, 2, …, *K*,
  1. estimate *π*_*k*_ by setting $\pi_{k}^{\left(t+1\right)}=\text{Card}\left(\chi_{k}^{\left(t+1\right)}\right)/n$, where Card(A) denotes the cardinality of the set *A*;
  2. apply the SIS-MCP algorithm [8, 24] to estimate the regression coefficients ***β**_k_* based on the samples assigned in $\chi_{k}^{\left(t+1\right)}$ and denote the estimate by $\beta_{k}^{\left(t+1\right)}$;
  3. estimate *σ*_*k*_ conditioned on the estimate $\beta_{k}^{\left(t+1\right)}$, i.e., set
    $$\begin{array}{c} \sigma_{k}^{\left(k+1\right)}=\sqrt{\frac{\sum_{i\in \chi_{k}^{\left(t+1\right)}}{\left(y_{i}-x_{i}\beta_{k}^{\left(t+1\right)}\right)}^{2}}{\text{Card}\left(\chi_{k}^{\left(t+1\right)}\right)-\text{Card}\left(\beta_{k}^{\left(t+1\right)}\right)-1}}, \end{array}$$ (5)
    where Card (***β**_k_*) denotes the number of nonzero elements in $\beta_{k}^{\left(t+1\right)}$.

In the SIS-MCP algorithm, the variables are first subject to a sure independence screening procedure [24], and then the survived variables are selected using the MCP method [8]. This algorithm has been implemented in the R-package *SIS*. This estimator maximizes the regularized conditional likelihood function as defined in (3), where the regularization function is given by the MCP penalty [8] in the subspace restricted by the sure independence screening procedure and ∞ otherwise. The consistency of the SIS-MCP estimator follows directly from [8, 24]. As shown in [21], such an estimator can be used in the ICC algorithm for achieving a consistent estimator for high-dimensional linear regression. Given an estimate of ***β**_k_*, we estimated *σ*_*k*_ using (5), for which the corresponding penalty function is 0, as it falls into the class of low-dimensional problems. Similarly, the penalty function was also set to zero in estimating *π*_*k*_’s. Following from [21], the sequence $\left\{\left(\pi_{k}^{\left(t\right)},\beta_{k}^{\left(t\right)},\sigma_{k}^{\left(t\right)}\right):k=1,2,\ldots ,K;t=1,2,\ldots \right\}$ will converge to the true parameter in probability as both *n* → ∞ and *t* → ∞. However, for a finite value of *n*, it will form a Markov chain which is almost surely ergodic and the average estimator (over *t* and with appropriate relabeling) is consistent.

In the above algorithm, we have assumed that *K* is known. To determine the value of *K*, we can use an average-BIC criterion which works as follows. First, we determine a set of *K* for consideration. Then for each value of *K* in the set, we run the ICC algorithm separately, obtain the sequences $\left\{\tau^{\left(t\right)}:t=1,2,\ldots \right\}$ and $\left\{\theta_{n}^{\left(t\right)}:t=1,2,\ldots ,T\right\}$, and calculate the BIC value for each *t* and their average. Mathematically, we have

$$\begin{array}{c} \hat{BIC}\left(K\right)=\frac{1}{T-t_{0}+1}\sum_{t=t_{0}+1}^{T}BIC_{K}\left(\tau^{\left(t\right)},\theta_{n}^{\left(t\right)}\right), \end{array}$$ (6)

where *t*_0_ denotes the burn-in steps of the Markov chains induced by ICC, *T* is the total number of iterations, and *BIC_K_*(***τ***^(*t*)^, ***θ***^(*t*)^) denotes the BIC value calculated based on the sample partition ***τ***^(*t*)^ and parameter estimate ***θ***^(*t*)^. The rationale underlying the average-BIC criterion can be justified as follows by viewing BIC as a value of negative log-posterior probability:

$$\begin{array}{c} \begin{array}{cc} -BIC(K) & =\text{log}\;(\sum_{\theta ,\tau }P\left(\theta ,\tau |K,D\right))=\text{log}\;(\sum_{\theta ,\tau }\frac{P(\theta ,\tau |K,D)}{P(\theta ,\tau )}P\left(\theta ,\tau \right))\\ & \geq \sum_{\theta ,\tau }P\left(\theta ,\tau \right)\;\text{log}\;(\frac{P(\theta ,\tau |K,D)}{P(\theta ,\tau )})\\ & =\sum_{\theta ,\tau }P\left(\theta ,\tau \right)\;\text{log}\;P\left(\theta ,\tau |K,D\right)-\sum_{\theta ,\tau }P\left(\theta ,\tau \right)\;\text{log}\;P\left(\theta ,\tau \right)\\ & \approx -\sum_{\theta ,\tau }P\left(\theta ,\tau \right)BIC_{K}\left(\tau ,\theta \right)-\sum_{\theta ,\tau }P\left(\theta ,\tau \right)\;\text{log}\;P\left(\theta ,\tau \right), \end{array} \end{array}$$

where D denotes the data, and the equality (in the second line) holds if P(θ,τ)=P(θ,τ|K,D). Further, by the asymptotic normality of the posterior distribution of ***θ*** (in the low-dimensional space restricted by the sure independence screening procedure), $\hat{BIC}\left(K\right)$ is approximately equivalent to BIC(K) in determining the value of *K* when both the sample size *n* and the number of iterations *T* become large.

### Clusterwise variable selection

The ICC algorithm proposed above leads to two interleaved Markov chains $\left\{\theta_{n}^{\left(t\right)}:t=1,2,\ldots \right\}$ and {***τ***^(*t*)^: *t* = 1, 2, …}. Therefore, different variables are selected at different iterations. How to aggregate the variables selected at different iterations into a single list remains an unresolved issue. To resolve this issue, we adopt the consensus clustering method [25–27], which works in the following procedure:

- Calculate a dissimilarity matrix *D* = (*d*_*ij*_) with
  $$\begin{array}{c} d_{ij}=T-t_{0}-\sum_{t=t_{0}+1}^{T}I\left(\tau_{i}^{\left(t\right)}=\tau_{j}^{\left(t\right)}\right), \end{array}$$ (7)
  where *I*(⋅) is an indicator whether or not sample *i* and sample *j* are assigned to the same cluster at iteration *t*.
- Cluster the samples into *K* clusters using a hierarchical clustering method, say, with the average linkage.
- Apply the SIS-MCP method to select variables for each cluster of samples separately.

The variables selected via this aggregation procedure are consistent, and its consistency follows directly from the consistency of the averaged ICC estimator.

### An illustrative example

To illustrate the performance of the proposed method, we consider an example which consists of 100 simulated datasets. Each dataset is independently generated according to (1) with *n* = 600, *p*_*n*_ = 2000, *K* = 3, *π*_1_ = *π*_2_ = *π*_3_ = 1/3, and $\sigma_{1}^{2}=\sigma_{2}^{2}=\sigma_{3}^{2}=1$. In simulations, we set *n*_1_ = *n*_2_ = *n*_3_ = 200, where *n*_*k*_ denotes the number of samples generated from component *k* of (1). For each value of *k*, ***β**_k_* consists of three nonzero elements which are all set to 3. To make the problem harder, we let ***β**_k_*’s share a common nonzero element and set all other nonzero elements to be exclusive. Each predictor ***x**_i_*, *i* = 1, 2, …, *p*, is generated from $N(\mu 1,I_{p_{n}})$, where $I_{p_{n}}$ is a *p*_*n*_-dimensional identity matrix, *μ***1** denotes a constant vector of *μ*, and *μ* is a random number generated from uniform(0,1),

The ICC algorithm was first applied to this example, which was started with a random assignment of the cluster membership for each of the samples. To measure the performance of the algorithm in both variable selection and sample clustering, we calculate the false selection rate and negative selection rate which are defined by

$$\begin{array}{c} fsr=\frac{FP}{TP+FP}\;nsr=\frac{FN}{TP+FN}, \end{array}$$ (8)

where TP, FP and FN refer to the true positive number, false positive number and false negative number, respectively, and they are defined via a binary decision table (see S1 Table). Note that both variable selection and sample clustering can be viewed as binary decision problems. For the former, it is to decide for each variable to be included in the model or not; and for the latter, it is to decide for each sample to be assigned to the correct cluster or not. In general, the smaller the values of *fsr* and *nsr* are, the better the performance of the method is. Other than *fsr* and *nsr*, we calculated for each cluster the estimation error of the regression coefficients, i.e., $\|{\hat{\beta }}_{k}-\beta_{k}\|=\sqrt{\sum_{i=1}^{p}{\left({\hat{\beta }}_{ki}-\beta_{ki}\right)}^{2}}$, where ${\hat{\beta }}_{k}$ denotes an estimate of ***β**_k_*.

S1 Fig shows the BIC statistics calculated along the path of $\left(\tau^{\left(t\right)},\theta_{n}^{\left(t\right)}\right)$, where it is assumed that the true value *K* = 3 is known. In the next subsection we explored the case that *K* is unknown. The ICC algorithm can converge very fast, usually with tens of iterations. For this example, it took about 50 iterations to converge, i.e., the Markov chain reaches equilibrium. To summarize the results of the run, we discarded the fist 100 iterations as the burn-in process and calculated the dissimilarity matrix, defined in (7), based on the next 400 iterations. Then the samples were re-clustered based on the dissimilarity matrix using a hierarchical clustering procedure with the average link, and variables were selected for each cluster using the SIS-MCP algorithm. The results are summarized in Table 1, which indicates that the algorithm works well for this example in both sample clustering and variable selection.

**Table 1:** Computational results of the ICC algorithm for the illustrative example, where fsr, nsr and $\|{\hat{\beta }}_{k}-\beta_{k}\|$ are calculated by averaging over 100 independent datasets with the standard deviation given in the parenthesis.

		Component 1	Component 2	Component 3
Variable Selection	*fsr*	0.01 (0.01)	0.02 (0.0198)	0 (0)
	*nsr*	0 (0)	0 (0)	0.01 (0.01)
	$\|{\hat{\beta }}_{k}-\beta_{k}\|$	0.4061 (0.1095)	0.4218 (0.1272)	0.3619 (0.1251)
Clustering	*fsr*	0.1522 (0.0094)	0.1287 (0.0104)	0.1499 (0.0135)
	*nsr*	0.1413 (0.0098)	0.1531 (0.0079)	0.1309 (0.0088)

For comparison, we have tried to apply the regularization method by [18] to this example. Unfortunately, the package *fmrs* cannot handle such a high-dimensional problem. For this reason, we considered another example in the next subsection where the dimension is set to be much lower.

To illustrate the prediction performance of ICC, we randomly selected 80% of the samples as the training data and the remaining for testing. The prediction results of the methods like Lasso, ridge, elastic net, and random forest were also included for comparison. Table 2 summarizes the computational results for 100 independent datasets.

**Table 2:** Prediction results of different methods for the illustrative example, where corr(*Y*_*test*_,${\hat{Y}}_{test}$) and RMSE(${\hat{Y}}_{test}$) are calculated by averaging over 100 independent datasets with the standard deviation given in the parenthesis.

	ICC	Lasso	Ridge	Elastic net	Random forest
corr(*Y*_*test*_,${\hat{Y}}_{test}$)	0.882 (0.097)	0.591 (0.086)	0.246 (0.128)	0.585 (0.092)	0.528 (0.089)
RMSE(${\hat{Y}}_{test}$)	3.076 (1.025)	5.333 (0.847)	6.576 (1.849)	5.378 (0.973)	5.738 (0.826)

## Results

### A comparison study

**Fig 1:**
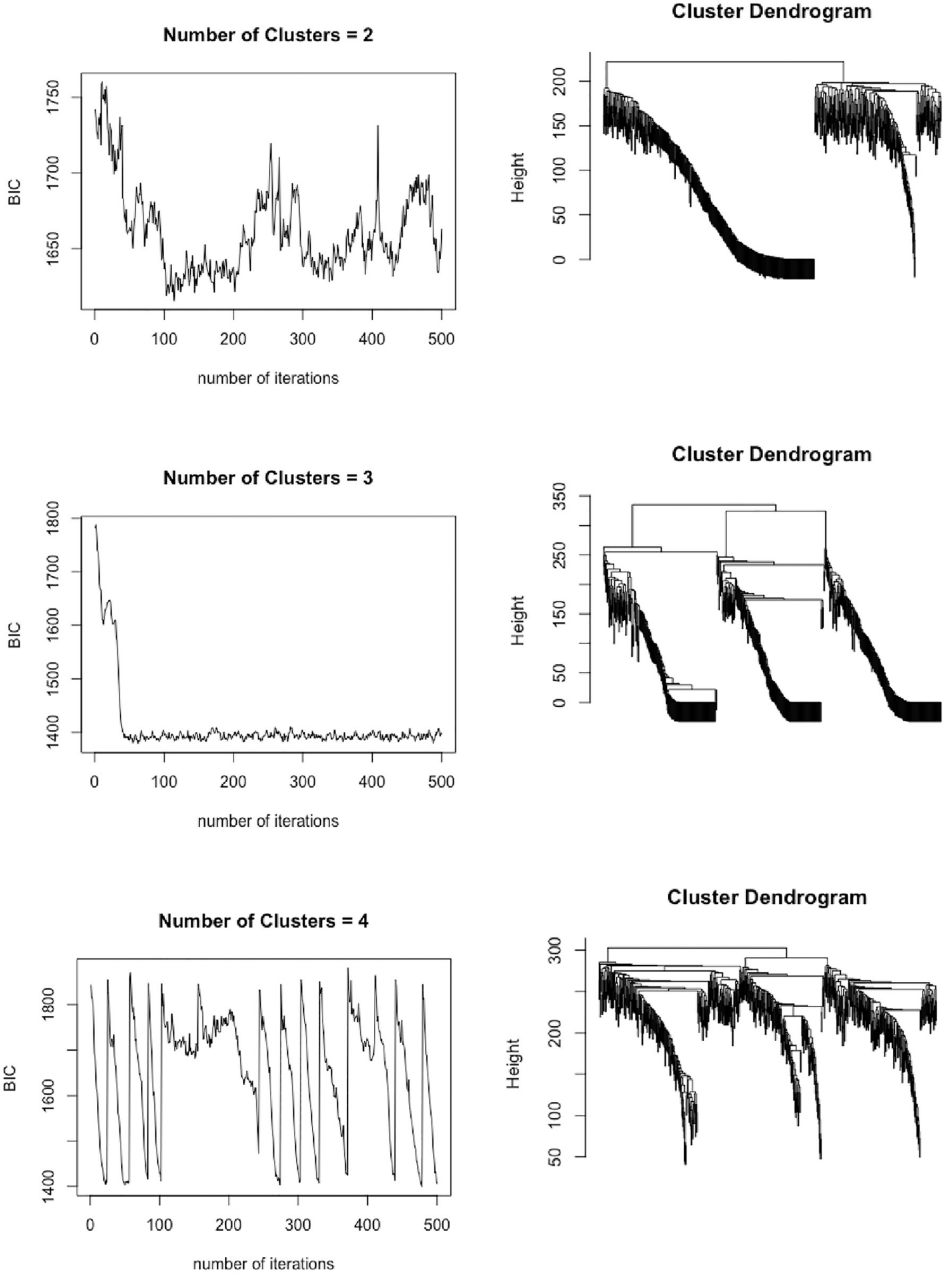
BIC paths and cluster dendrograms produced by ICC. The BIC paths and cluster dendrograms produced by ICC with *K* = 2, 3, 4, where each cluster dendrogram was produced using a hierarchical clustering procedure (with the average link) based on the dissimilarity matrix calculated along the corresponding BIC path after discarding the first 100 iterations as the burn-in process.

To make the package *fmrs* work, we independently generated 10 datasets as in the last subsection except for that the dimension *p*_*n*_ was reduced to 200. For each dataset, we tried three different values of *K* = 2, 3 and 4; and for each value of *K*, we ran the ICC algorithm for 500 iterations, where the first 100 iterations were discarded for the burn-in process and ***τ***^(*t*)^’s collected from the remaining 400 iterations were used for computing the dissimilarity matrix *D*. Each run took about 10 CPU minutes on a computer of 2.60GHz. Fig 1 shows the BIC paths generated by ICC with *K* = 2, 3 and 4 for one dataset. According to the average-BIC criterion, we can easily determine that *K* = 3. The corresponding cluster dendrogram shows that there is a clear cut between three clusters of the samples. Table 3 summarizes the computational results for the 10 datasets.

**Table 3:** Comparison of the ICC and regularization methods, where fsr, nsr and $\|{\hat{\beta }}_{k}-\beta_{k}\|$ are calculated by averaging over 10 independent datasets with the standard deviation given in the parenthesis. The regularization methods were implemented with the Lasso, SCAD and MCP penalties.

ICC		Component 1	Component 2	Component 3
Variable Selection	*fsr*	0.0167 (0.0527)	0 (0)	0.0286 (0.0904)
	*nsr*	0 (0)	0 (0)	0 (0)
	$\|{\hat{\beta }}_{k}-\beta_{k}\|$	0.4128 (0.1712)	0.4437 (0.1743)	0.4511 (0.2201)
Clustering	*fsr*	0.2032 (0.0231)	0.2218 (0.0402)	0.1956 (0.0391)
	*nsr*	0.1965 (0.0433)	0.198 (0.0572)	0.227 (0.0493)
CPU(m)		10		
fmrs-Lasso		Component 1	Component 2	Component 3
Variable Selection	*fsr*	0.32773 (0.2455)	0.0429 (0.1355)	0.05 (0.1581)
	*nsr*	0.52 (0.3676)	0.78 (0.3824)	0.94 (0.1897)
	$\|{\hat{\beta }}_{k}-\beta_{k}\|$	8.778 (0.9603)	9.273 (1.3057)	9.884 (0.3668)
Clustering	*fsr*	0.6989 (0.1029)	0.8044 (0.2192)	0.7847 (0.2524)
	*nsr*	0.3095 (0.4607)	0.7755 (0.3918)	0.8925 (0.3067)
CPU(m)		6		
fmrs-SCAD		Component 1	Component 2	Component 3
Variable Selection	*fsr*	0.1650 (0.2371)	0.0556 (0.1757)	0.0775 (0.1635)
	*nsr*	0.56 (0.3098)	0.8 (0.2828)	0.749 (0.3458)
	$\|{\hat{\beta }}_{k}-\beta_{k}\|$	7.927 (2.8518)	9.35 (0.8100)	9.749 (0.5852)
Clustering	*fsr*	0.5514 (0.2312)	0.7303 (0.3041)	0.8622 (0.1799)
	*nsr*	0.4405 (0.4207)	0.664 (0.4117)	0.8485 (0.3086)
CPU(m)		5		
fmrs-MCP		Component 1	Component 2	Component 3
Variable Selection	*fsr*	0.09 (0.1912)	0.05 (0.1581)	0.1333 (0.2194)
	*nsr*	0.54 (0.1897)	0.74 (0.2119)	0.64 (0.3239)
	$\|{\hat{\beta }}_{k}-\beta_{k}\|$	7.475 (0.8900)	8.253 (1.7369)	7.539 (3.2272)
Clustering	*fsr*	0.5427 (0.0862)	0.5652 (0.1118)	0.6074 (0.1164)
	*nsr*	0.5305 (0.3691)	0.6225 (0.3550)	0.5455 (0.3529)
CPU(m)		5		

For comparison, the regularization method in [18] were applied to this example with three different penalty functions, including Lasso, SCAD and MCP. The respective results and CPU costs were also included in Table 3. The comparison indicates that the proposed method has made a drastic improvement over the regularization method in both variable selection and sample clustering, while having a comparable CPU cost with the existing regularization method.

Finally we note that the ICC algorithm can converge very fast, usually within 50 iterations. To be safe, we set the number of burn-in iterations to 100 and then continue to run for 400 iterations for sample collection. Such a 500-iteration run has been excessively long for ICC.

### Drug sensitivity prediction and sensitive gene selection

The Cancer Cell Line Encyclopedia (CCLE) dataset consisted of 8-point dose-response curves for 24 chemical compounds across over 400 cell lines. For different chemical compounds, the numbers of cell lines are slightly different. For each cell line, it consisted of the expression data of 18,926 genes. The dataset is publicly available at www.broadinstitute.org/ccle. We used the area under the dose-response curve, which is also termed as activity area, to measure the sensitivity of a drug for each cell line. Compared to other measurements, such as *IC*_50_ and *EC*_50_, the activity area could capture the efficacy and potency of a drug simultaneously. To pre-process the data, for each drug, we first applied a model-free feature screening method proposed in [28] to reduce the number of candidate genes to *p*_*n*_ = 500 and then divided the cell lines to two parts, the first 80% of the cell lines used for training and the remaining 20% of the cell lines used for test (in the order published at the CCLE website).

The underlying scientific motivation for this study is that cancer is a complex disease and it can have significant heterogeneity in response to treatments. Therefore, the mixture regression is potentially appropriate for modeling such heterogeneous data. We note that the drug-sensitive genes, that are identified by the proposed method based on the CCLE data, may differ from those genes that respond to the drug. To truly identify the genes that respond to the drug, i.e., those whose expression changes with drug treatments or dose levels, statistically we have to take the drug level as covariates and the gene expression as the response variable.

For each dataset, we tried four different values of *K* = 1, 2, 3 and 4. For the case *K* = 1, the ICC algorithm is simply reduced to the SIS-MCP algorithm for conventional high-dimensional linear regression. In this case, it only needs to run for a single iteration. For *K* = 2, 3 or 4, we ran the ICC algorithm for 500 iterations. As in simulation studies, we discarded the first 100 iterations for the burn-in process and used the remaining 400 iterations were for inference. Each run costs about 15 CPU minutes on a computer of 2.60GHz. The computational results were summarized in Table 4, where the number of clusters for each drug was determined according to the average-BIC criterion. In addition to the value of *K*, Table 4 also reports the total number of genes selected by the mixture regression, the correlation coefficient corr(*Y*_train_, ${\hat{Y}}_{\text{train}}$), and the correlation coefficient corr(*Y*_test_, ${\hat{Y}}_{\text{test}}$), where ${\hat{Y}}_{\text{train}}$ and ${\hat{Y}}_{\text{test}}$ denote the fitted and predicted response, respectively. The genes selected by the mixture regression for each drug was reported in S4 Table. In prediction, we first identify the cluster that the new sample most likely belongs to according to the distribution given in (4) and then make the prediction based on the regression model learned for that cluster. To show the advantage of the mixture regression model, we have also included in Table 4 the results with *K* = 1 for all drugs. Among the 24 drugs, there are 20 drugs that prefer the mixture regression model according to the average-BIC criterion. For these 20 drugs, the mixture regression model has made a drastic improvement over the single regression model in both fitting and prediction. To make this conclusion more concrete, we also conducted random shuffling and a 5-fold cross validation on the CCLE dataset, the results are included in S2 and S3 Tables.

**Table 4:** Comparison of the single component regression model and mixture regression model for the CCLE dataset, where #gene denotes the total number of different genes selected by the model.

Drug	Single regression Model					Mixture regression Model					
	#gene	corr(*Y*_train_,${\hat{Y}}_{\text{train}}$)	corr(*Y*_test_,${\hat{Y}}_{\text{test}}$)	RMSE(${\hat{Y}}_{\text{train}}$)	RMSE(${\hat{Y}}_{\text{test}}$)	K	#gene	corr(*Y*_train_,${\hat{Y}}_{\text{train}}$)	corr(*Y*_test_,${\hat{Y}}_{\text{test}}$)	RMSE(${\hat{Y}}_{\text{train}}$)	RMSE(${\hat{Y}}_{\text{test}}$)
17-AAG	15	0.655	0.469	0.785	0.937	1	15	0.655	0.469	0.785	0.937
AEW541	8	0.555	0.289	0.503	0.609	2	9	0.835	0.73	0.325	0.433
AZD0530	12	0.541	0.368	0.662	0.714	2	5	0.819	0.816	0.439	0.448
AZD6244	13	0.71	0.637	0.651	0.709	2	10	0.855	0.783	0.399	0.387
Erlotinib	1	0.403	0.207	0.810	0.733	2	7	0.814	0.759	0.528	0.489
Irinotecan	4	0.769	0.647	0.724	0.869	3	5	0.921	0.873	0.443	0.558
L-685458	3	0.586	0.444	0.449	0.485	3	7	0.924	0.888	0.209	0.248
LBW242	2	0.341	0.3	0.842	0.887	3	11	0.919	0.862	0.501	0.543
Lapatinib	1	0.489	0.351	0.563	0.655	2	5	0.871	0.819	0.318	0.399
Nilotinib	2	0.548	0.272	0.675	0.632	2	7	0.891	0.72	0.401	0.453
Nutlin-3	1	0.322	0.302	0.822	0.839	3	23	0.925	0.86	0.507	0.518
PD-0325901	17	0.756	0.693	0.562	0.593	4	14	0.81	0.798	0.363	0.371
PD-0332991	1	0.492	0.3	0.542	0.640	1	1	0.492	0.3	0.542	0.640
PF2341066	2	0.466	0.133	0.568	0.619	3	4	0.909	0.802	0.389	0.408
PHA-665752	6	0.484	0.129	0.477	0.511	4	11	0.744	0.783	0.355	0.413
PLX4720	1	0.453	0.436	0.493	0.783	2	3	0.83	0.724	0.397	0.598
Paclitaxel	10	0.629	0.584	1.001	1.081	4	6	0.759	0.682	0.842	0.955
Panobinostat	3	0.656	0.5	0.861	0.893	4	13	0.88	0.834	0.716	0.734
RAF265	10	0.574	0.392	0.601	0.737	1	10	0.574	0.392	0.601	0.737
Sorafenib	1	0.493	0.209	0.671	0.833	4	21	0.931	0.774	0.518	0.545
TAE684	3	0.495	0.263	0.737	0.816	2	10	0.837	0.792	0.462	0.515
TKI258	2	0.461	0.039	0.553	0.556	3	9	0.815	0.732	0.349	0.373
Topotecan	5	0.691	0.529	0.905	1.080	4	8	0.829	0.751	0.708	0.810
ZD-6474	8	0.473	0.24	0.889	0.973	1	8	0.473	0.24	0.889	0.973

**Fig 2:**
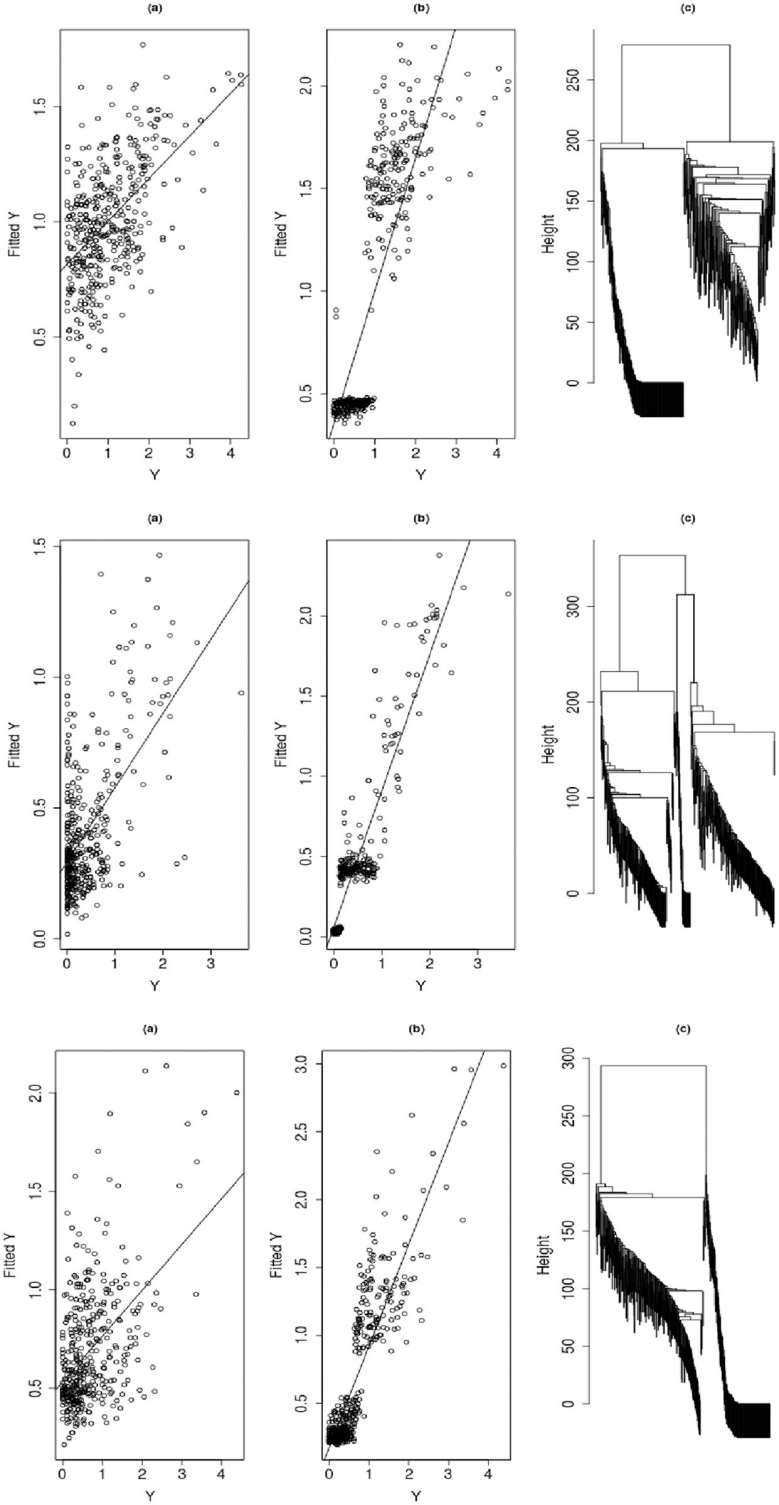
Comparison of the single component and mixture regression models for training data fitting. The left column is for the single regression model, the middle column is for the mixture regression model, and the right column is the cluster dendrogram produced by the mixture regression model; the top, middle and lower panels are for the drugs AZD0530, L-685458 and Lapatinib, respectively.

**Fig 3:**
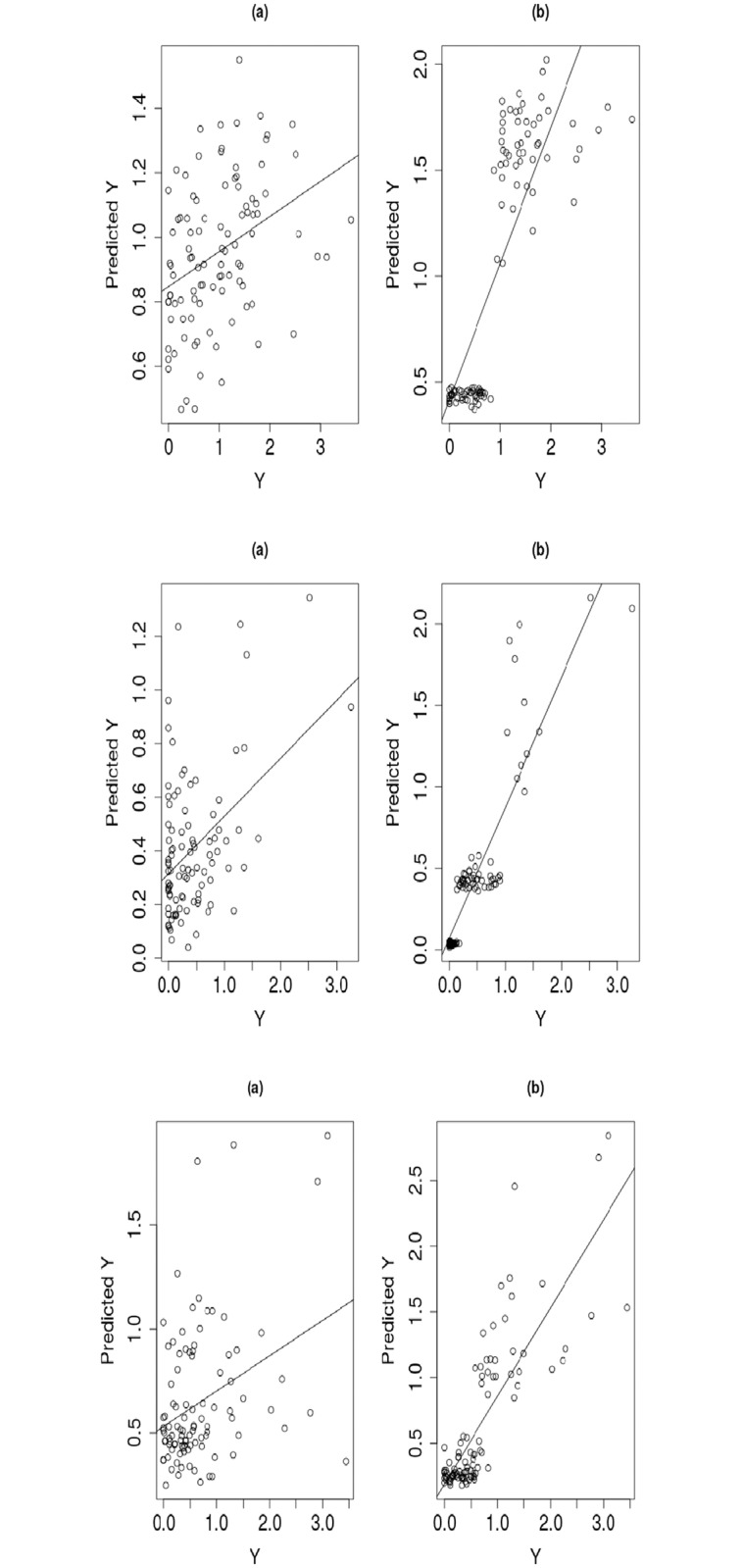
Comparison of the single component and mixture regression models for test data prediction. The left column is for the single component regression model, and the right column is for the mixture regression model; the top, middle and lower panels are for the drugs AZD0530, L-685458 and Lapatinib, respectively.

To visualize the detailed fitting and prediction performance of the mixture regression model, we show in Figs 2 and 3 some scatter plots and cluster dendrograms with the drugs AZD0530, L-685458 and Lapatinib as examples. In fitting, the values of corr(*Y*_train_,${\hat{Y}}_{\text{train}}$) of the three drugs have been improved by the mixture regression model from 0.541, 0.586 and 0.489 to 0.819, 0.924 and 0.871, respectively. In prediction, the values of corr(*Y*_test_,${\hat{Y}}_{\text{test}}$) of the three drugs have been improved by the mixture regression model from 0.289, 0.444 and 0.351 to 0.73, 0.888 and 0.819, respectively.

In terms of gene selection, we find that for most drugs, the genes selected by the mixture regression model are quite consistent with our existing knowledge. For example, for both drug AZD6244 and PD-0325901, the gene SPRY2 was selected by the mixture regression model. It is known that SPRY2 is an inhibitor of mitogen-activated protein kinase signaling, and it has been recognized as the top sensitive gene to the two drugs [10, 29, 30]. [30] reported that the gene DUSP6 is one of the key genes under MEK function control, while MEK is the target gene of AZD6244. In our study, this gene was selected by the mixture regression model but not by the single regression model. For the drug Topotecan and Irinotecan, the gene SLFN11 was selected as the top drug sensitive gene. Both [3] and [31] reported that SLFN11 is a predictive biomarker for these two drugs. For the drug Lapatinib, the gene ERBB2 was selected by the mixture regression model but not by the single regression model. Both [32] and [33] reported that the expression level of ERBB2 is predictive for the treatment effect of Lapatinib. For the drug Paclitaxel, the gene BCL2L1 was again selected by the mixture regression model only. In the literature, [34] reported that the gene BCL2L1 is predictive for the treatment effect of Paclitaxel.

We note that for some drugs, including TKI258, PHA-665752 and Topotecan, there are some clusters for which no genes were selected. We have made a detailed exploration of these clusters. The reason is that these clusters are too small, each consisting of 2 samples only, and thus no genes were selected. Merging them to other clusters is possible, but this will lead to a slight increase in the average-BIC value. In general, for two partitions with similar average-BIC values, the prediction will not be much affected.

For a thorough comparison, we have also applied elastic net, ridge regression, support vector regression and random forest to this example, which have been implemented in the R package *glmnet*, *glmnet*, *e1071* and *randomForest*, respectively. For elastic net, we let the *l*_1_-penalty and the *l*_2_-penalty to be equally weighted, and let the regularization parameter determined via cross-validation. For ridge regression, we determine the regularization parameter via cross-validation. For support vector regression, we have tried all possible combinations of the kernels (linear, sigmoid, radial and polynomial) and regression types (eps-regression and nu-regression). For random forest, we run the package under the default setting. We have also tried different numbers of trees, but less favorable results were produced. The results were summarized in S5 Table.

**Fig 4:**
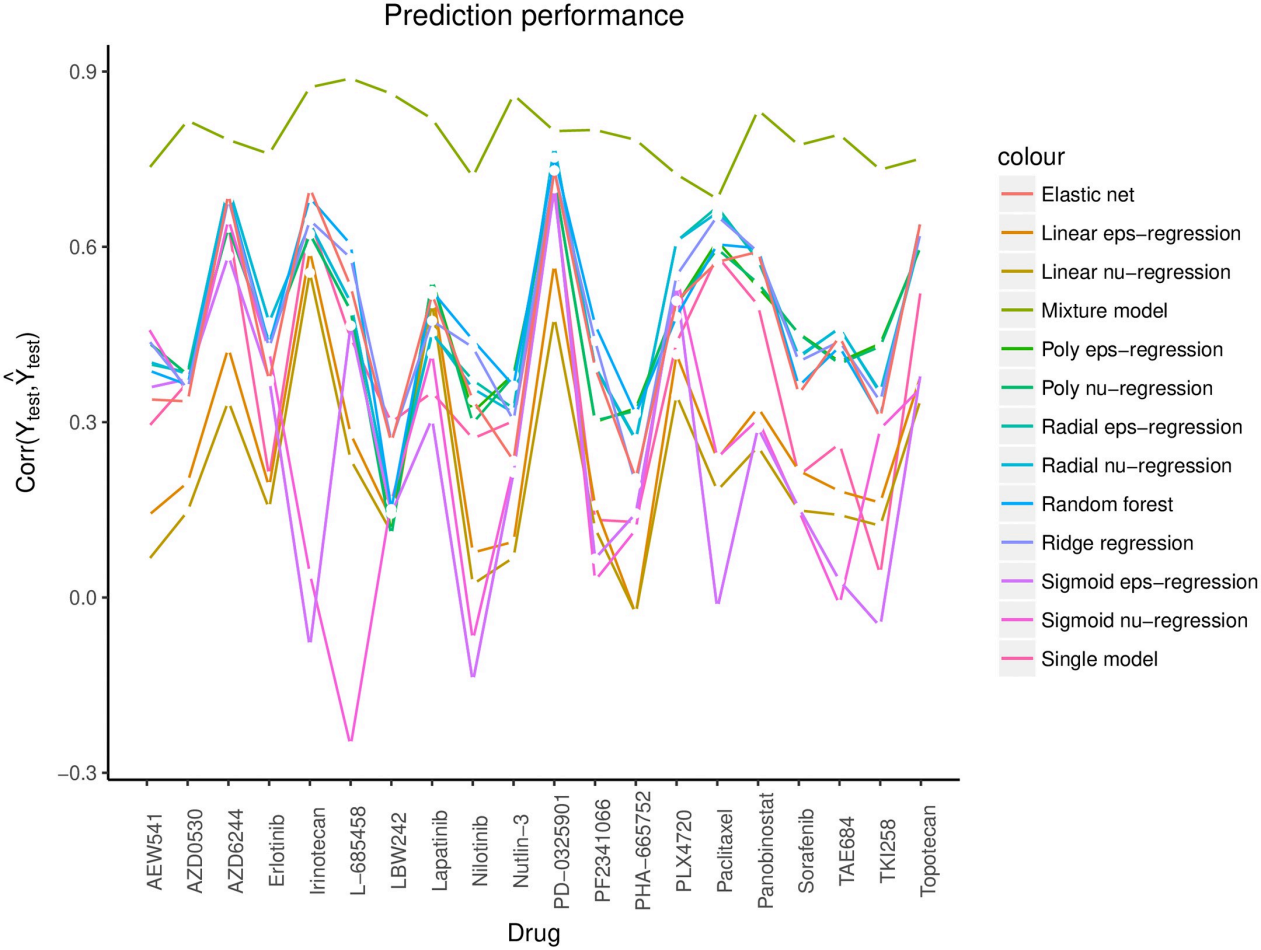
Prediction performance comparison. Comparison of the prediction performance (measured by corr(*Y*_test_, ${\hat{Y}}_{\text{test}}$)) of the mixture regression model with support vector regression, random forest, ridge regression, elastic net, and SIS-MCP regression (i.e., single model regression).

As a summary, we show in Fig 4 the values of corr(*Y*_test_,${\hat{Y}}_{\text{test}}$) produced by support vector regression, random forest, ridge regression, elastic net, SIS-MCP regression (i.e., single model regression), and mixture regression for the 20 drugs that prefer the mixture regression model. The plot indicates that the mixture model has made a drastic improvement in prediction over all other competitive methods for these drugs. To assess the significance of the results, we applied Fisher’s transformation to each of the correlation coefficients between *Y*_test_ and ${\hat{Y}}_{\text{test}}$; that is, we define the prediction *z*-score by

$$\begin{array}{c} Z=0.5\;\text{log}\;(\frac{1+r}{1-r}), \end{array}$$

where *r* denotes the correlation coefficient. Following the standard statistical theory, *Z* is approximately normally distributed with a standard deviation of $1/\sqrt{N-3}$, where *N* denotes the number of samples used in calculating the correlation coefficient *r*. Based on the prediction *z*-scores, we conducted paired *t*-tests for each of the competitive methods versus the mixture regression method and reported the *p*-values in Table 5. The tests were under two scenarios, with all *N* = 24 drugs and with only the *N* = 20 drugs for which the mixture regression is preferred. Under both scenarios, the mixture regression method shows highly significant improvement in prediction over the competitive methods. These results imply that population heterogeneity is the key to the success of the proposed method. If the population is homogeneous (i.e., a single component regression model is preferred), the regularized linear regression might not be the best method for drug sensitivity prediction. In this case, both support vector regression and random forest tend to work better than regularized linear regression.

**Table 5:** *p*-values produced by the paired *t*-test for each of the competitive methods versus the mixture regression.

	linear & eps-reg	linear & nu-reg	radial & eps-reg	radial& nu-reg	poly& eps-reg	poly &nu-reg	sigmoid &eps-reg	sigmoid & nu-reg	random forest	ridge	elastic net	Single
20 Drugs	9.88e-13	6.92e-14	3.69e-8	3.49e-8	2.58e-9	2.34e-9	1.02e-10	4.50e-9	6.72e-9	1.01e-10	4.17e-11	9.88e-13
24 Drugs	1.15e-9	2.00e-10	1.26e-5	1.31e-5	1.32e-6	1.23e-6	9.48e-8	5.82e-7	6.89e-6	4.26e-7	9.37e-8	2.30e-8

## Discussion

The proposed method can be extended in various ways. In the current implementation, the features are selected at each iteration using the SIS-MCP algorithm. Many other algorithms can be used in place of SIS-MCP, e.g., SIS-SCAD [7, 24] and rLasso [9]. However, the Lasso algorithm [6] might not work here, which, as pointed out in [21], will lead to a biased estimator if the parameter space is unbounded. In addition, the ICC algorithm requires the estimates $\theta_{n}^{\left(t\right)}$’s to be accurate enough at each iteration. To be more precise, $\theta_{n}^{\left(t\right)}$’s need to be uniformly consistent with respect to $\theta_{*}^{\left(t\right)}$’s. For the model considered in the paper, a high-quality high-dimensional regression estimation procedure is essential for achieving such a goal. It is known that the performance of the penalized likelihood methods, which include MCP and SCAD, tend to deteriorate as the dimension increases, see [35] for more discussions on this issue. To address this issue, a blockwise consistency method by [36] might be used to further improve the performance of the MCP and SCAD method when the number of features is ultra-high.

To determine the value of *K*, i.e., the number of clusters, we proposed the average-BIC criterion. With the same reasoning, an average EBIC criterion can be defined based on the EBIC statistic [37], and it might work better for high-dimensional problems. Finally, we would like to say that the proposed method is general, which can work with any types of features, e.g., genomic features, clinical features and demographical features.

## Conclusion

We have proposed a mixture regression model-based method for drug sensitivity prediction. The proposed method has explicitly addressed two fundamental issues in drug sensitivity prediction, namely, population heterogeneity and feature selection pertaining to each subpopulation. The mixture regression model is estimated using the ICC algorithm, which can lead to a consistent estimator for the mixture regression model. In addition, we have proposed an average-BIC criterion for determining the number of components for the mixture regression model. The proposed method was applied to the CCLE dataset, and the numerical results indicate that the proposed method has made a drastic improvement over the existing ones, such as random forest, support vector regression, and regularized linear regression, in both response prediction and feature selection.

## Supporting information

S1 Table Outcomes of binary decision. (PDF)

S2 Table Results of single component regression. The results of single component regression with random shuffling and 5-fold cross validation on the CCLE dataset, where the results are averaged over the 5-fold runs with the standard deviation included in the parenthesis.
(PDF)

S3 Table Results of mixture regression. The results of mixture regression with random shuffling and 5-fold cross validation on the CCLE dataset, where the results are averaged over the 5-fold runs with the standard deviation included in the parenthesis.
(PDF)

S4 Table Drug sensitive genes. The drug sensitive genes selected by the ICC algorithm with the mixture regression model for the CCLE dataset: “–” indicates that no genes were selected for that cluster of samples.
(PDF)

S5 TablePrediction performance comparison.The values of corr(*Y*_train_,${\hat{Y}}_{\text{train}}$) and corr(*Y*_test_,${\hat{Y}}_{\text{test}}$) produced by support vector regression ({linear, radial, polynomial, sigmoid} ×{eps-regression, nu-regression}), random forest, ridge regression and elastic net for the CCLE dataset.(PDF)

S1 Fig BIC path. The BIC path produced by the ICC algorithm during the first 2000 iterations.
(TIFF)
