# Supplementary material for: Drug sensitivity prediction with high-dimensional mixture regression
Source: PLoS One. 2019 Feb 27;14(2):e0212108. doi: 10.1371/journal.pone.0212108 (PMC6392252; doi:10.1371/journal.pone.0212108)
Supplement: S1 Table — (PDF) [file pone.0212108.s001.pdf]

Table S1

	$A_{ij} = 1$	$A_{ij} = 0$
$\hat{A}_{ij} = 1$	True Positive (TP)	False Positive (FP)
$\hat{A}_{ij} = 1$	False Negative (FN)	True Negative (TN)
