# Supplementary material for: Drug sensitivity prediction with high-dimensional mixture regression
Source: PLoS One. 2019 Feb 27;14(2):e0212108. doi: 10.1371/journal.pone.0212108 (PMC6392252; doi:10.1371/journal.pone.0212108)
Supplement: S2 Table — The results of single component regression with random shuffling and 5-fold cross validation on the CCLE dataset, where the results are averaged over the 5-fold runs with the standard deviation included in the parenthesis. (PDF) [file pone.0212108.s002.pdf]

Table S2

Drug	#gene	corr(Y_{train},$\widehat{Y}_{\text{train}}$)	RMSE($\widehat{Y}_{\text{train}}$)	corr(Y_{test},$\widehat{Y}_{\text{test}}$)	RMSE($\widehat{Y}_{\text{test}}$)
17-AAG	13.2 (2.86)	0.621 (0.020)	0.818 (0.020)	0.481 (0.044)	0.883 (0.019)
AEW541	6.8 (3.31)	0.487 (0.068)	0.539 (0.021)	0.340 (0.095)	0.576 (0.031)
AZD0530	9.4 (2.70)	0.516 (0.043)	0.688 (0.022)	0.352 (0.084)	0.720 (0.039)
AZD6244	11 (2.24)	0.691 (0.049)	0.713 (0.038)	0.598 (0.027)	0.734 (0.044)
Erlotinib	2.8 (1.64)	0.412 (0.035)	0.865 (0.046)	0.231 (0.039)	0.817 (0.021)
Irinotecan	3.4 (1.67)	0.799 (0.053)	0.782 (0.053)	0.654 (0.019)	0.855 (0.030)
L-685458	2.8 (1.23)	0.512 (0.042)	0.496 (0.081)	0.428 (0.057)	0.522 (0.028)
LBW242	2.8 (1.30)	0.383 (0.061)	0.813 (0.074)	0.311 (0.067)	0.883 (0.022)
Lapatinib	1.6 (0.89)	0.452 (0.039)	0.593 (0.052)	0.381 (0.075)	0.710 (0.034)
Nilotinib	3.2 (1.30)	0.571 (0.072)	0.599 (0.094)	0.282 (0.053)	0.619 (0.034)
Nutlin-3	2.4 (1.65)	0.376 (0.047)	0.841 (0.033)	0.324 (0.056)	0.816 (0.085)
PD-0325901	15.8 (2.87)	0.722 (0.058)	0.610 (0.092)	0.691 (0.012)	0.603 (0.045)
PD-0332991	1.6 (0.55)	0.482 (0.029)	0.572 (0.056)	0.445 (0.066)	0.558 (0.059)
PF2341066	2.8 (1.10)	0.479 (0.038)	0.592 (0.093)	0.174 (0.036)	689 (0.046)
PHA-665752	7.2 (2.83)	0.481 (0.032)	0.491 (0.054)	0.107 (0.022)	0.532 (0.085)
PLX4720	1.6 (0.89)	0.465 (0.027)	0.509 (0.052)	0.472 (0.058)	0.715 (0.082)
Paclitaxel	8.2 (1.64)	0.649 (0.039)	0.974 (0.018)	0.551 (0.083)	1.037 (0.056)
Panobinostat	3.8 (1.58)	0.614 (0.064)	0.835 (0.037)	0.488 (0.028)	0.895 (0.016)
RAF265	12.4 (2.21)	0.602 (0.052)	0.599 (0.041)	0.334 (0.092)	0.701 (0.039)
Sorafenib	1.4 (0.95)	0.523 (0.056)	0.618 (0.034)	0.218 (0.024)	0.816 (0.082)
TAE684	3.4 (2.07)	0.467 (0.017)	0.763 (0.052)	0.285 (0.039)	0.801 (0.055)
TKI258	3.6 (1.67)	0.479 (0.038)	0.552 (0.028)	0.050 (0.013)	0.589 (0.026)
Topotecan	4.8 (1.04)	0.624 (0.045)	0.898 (0.071)	0.513 (0.064)	1.001 (0.092)
ZD-6474	6.8 (2.36)	0.481 (0.032)	0.816 (0.027)	0.294 (0.127)	0.869 (0.063)
