# Supplementary material for: Drug sensitivity prediction with high-dimensional mixture regression
Source: PLoS One. 2019 Feb 27;14(2):e0212108. doi: 10.1371/journal.pone.0212108 (PMC6392252; doi:10.1371/journal.pone.0212108)
Supplement: S3 Table — The results of mixture regression with random shuffling and 5-fold cross validation on the CCLE dataset, where the results are averaged over the 5-fold runs with the standard deviation included in the parenthesis. (PDF) [file pone.0212108.s003.pdf]

Table S3

Drug	# of clusters	#gene	corr(Y_{train},$\widehat{Y}_{\text{train}}$)	RMSE($\widehat{Y}_{\text{train}}$)	corr(Y_{test},$\widehat{Y}_{\text{test}}$)	RMSE($\widehat{Y}_{\text{test}}$)
17-AAG	1 (0)	13.2 (2.86)	0.621 (0.020)	0.818 (0.020)	0.481 (0.044)	0.883 (0.019)
AEW541	2 (0)	9.6 (1.04)	0.859 (0.032)	0.346 (0.057)	0.707 (0.079)	0.382 (0.082)
AZD0530	2 (0)	4.8 (1.99)	0.827 (0.018)	0.482 (0.024)	0.822 (0.087)	0.412 (0.075)
AZD6244	2 (0)	11.2 (2.48)	0.827 (0.047)	0.401 (0.055)	0.759 (0.062)	0.426 (0.053)
Erlotinib	2 (0)	5.8 (2.59)	0.795 (0.042)	0.588 (0.037)	0.763 (0.082)	0.491 (0.071)
Irinotecan	2.8 (0.45)	5 (1.58)	0.901 (0.038)	0.489 (0.064)	0.884 (0.060)	0.526 (0.055)
L-685458	3 (0)	8.2 (2.01)	0.901 (0.047)	0.239 (0.027)	0.874 (0.058)	0.301 (0.055)
LBW242	3 (0)	12.2 (3.27)	0.896 (0.052)	0.520 (0.034)	0.826 (0.091)	0.601 (0.086)
Lapatinib	2 (0)	4.8 (1.30)	0.839 (0.049)	0.322 (0.042)	0.799 (0.057)	0.402 (0.048)
Nilotinib	2 (0)	6.2 (1.69)	0.767 (0.093)	0.498 (0.038)	0.733 (0.062)	0.475 (0.053)
Nutlin-3	3 (0)	20.8 (3.58)	0.912 (0.042)	0.481 (0.026)	0.827 (0.045)	0.486 (0.052)
PD-0325901	4 (0)	12.4 (2.48)	0.823 (0.034)	0.327 (0.045)	0.769 (0.084)	0.421 (0.074)
PD-0332991	1 (0)	1.6 (0.55)	0.482 (0.029)	0.572 (0.056)	0.445 (0.066)	0.558 (0.059)
PF2341066	3 (0)	4.4 (1.90)	0.849 (0.058)	0.417 (0.025)	0.812 (0.042)	0.473 (0.058)
PHA-665752	3.6 (0.55)	9.8 (3.35)	0.720 (0.051)	0.383 (0.051)	0.772 (0.098)	0.424 (0.086)
PLX4720	2 (0)	3.2 (0.45)	0.810 (0.052)	0.411 (0.027)	0.736 (0.052)	0.578 (0.049)
Paclitaxel	4 (0)	5.4 (1.55)	0.768 (0.069)	0.884 (0.053)	0.653 (0.097)	0.989 (0.085)
Panobinostat	4 (0)	12 (1.83)	0.873 (0.045)	0.733 (0.049)	0.753 (0.065)	0.781 (0.05)
RAF265	1 (0)	12.4 (2.21)	0.602 (0.052)	0.599 (0.041)	0.334 (0.092)	0.701 (0.039)
Sorafenib	3.8 (0.45)	21 (7.58)	0.909 (0.045)	0.533 (0.062)	0.791 (0.077)	0.686 (0.055)
TAE684	2 (0)	8.8 (2.13)	0.823 (0.034)	0.485 (0.085)	0.749 (0.036)	0.585 (0.045)
TKI258	3 (0)	9.2 (1.85)	0.798 (0.024)	0.381 (0.048)	0.746 (0.047)	0.398 (0.033)
Topotecan	4 (0)	7.4 (0.97)	0.774 (0.083)	0.794 (0.076)	0.738 (0.082)	0.842 (0.059)
ZD-6474	1 (0)	6.8 (2.36)	0.481 (0.032)	0.816 (0.027)	0.294 (0.127)	0.869 (0.063)
