# Supplementary material for: Drug sensitivity prediction with high-dimensional mixture regression
Source: PLoS One. 2019 Feb 27;14(2):e0212108. doi: 10.1371/journal.pone.0212108 (PMC6392252; doi:10.1371/journal.pone.0212108)
Supplement: S4 Table — The drug sensitive genes selected by the ICC algorithm with the mixture regression model for the CCLE dataset: “–” indicates that no genes were selected for that cluster of samples. (PDF) [file pone.0212108.s004.pdf]

Table S4

Drug	Cluster	Selected Genes
17-AAG	1	NQO1, RTF1, ZNF610, UNC5A, CDH6, RPUSD4, SEC23IP, ZADH2, SREBF1, MMP24, GFRA1, GPC4, MAP7D2, OGDHL, G3BP2
AEW541	1	LOC151534, LOC100506319, HCCS, CNNM3, UBAC2, SP1, MID2
	2	SETDB1, ALCAM
AZD0530	1	APOO, THEM4, SRPX
	2	ANKRD44, NR2F6
AZD6244	1	SPRY2, LOC146880
	2	ASPRY2, CYR61, TMC6, PIWIL4, BEND7, HIVEP1, HSF2BP, COL9A3, DUSP6
Erlotinib	1	NMI, HES1, GJB6, BTBD11, LAD1, EFHC1
	2	LAD1
Irinotecan	1	ARHGAP19, CTSD
	2	SLFN11, KIF21B
	3	PDIK1L
L-685458	1	LINC00528
	2	EPHB4, LHFPL2, MYL9, ANXA2, HECA
	3	HMGB2
LBW242	1	CNTROB, STAT4, MTMR7, RHOV, ZC3HAV1
	2	SLC35A2, SLC35F5
	3	TGFB3, AGPAT2, RIPK1, BST2
Lapatinib	1	C2orf15, TRIM29, P2RY2
	2	ERBB2, IKBIP
Nilotinib	1	SRBD1, PEX11G, BCL6, HIST1H3F, CHST12
	2	TRAF3IP2-AS1, NUP54
Nutlin-3	1	APIAR, PRR14,TNFSF15, PUS10, DMRT3,FAM189B,MPP2,GNB3
	2	SCAND2, DNAJB14, PDIA3, RPS27L, ARID3A, C1orf182, ENTPD3, CCNY, MMAA, CCNG1, ZMAT3
	3	IL12B, EPC1, CDC14A, RAB5C
PD-0325901	1	SPRY2, TMC6, RNF125, DUSP6, LYZ, COL9A3, ABHD2
	2	LYZ, DCAF4, C6orf203, GRIN2A, MRP63
	3	TGFA
	4	SCARA3, FGD1
PD-0332991	1	CMFG
PF2341066	1	GHRLOS2
	2	IL13RA1
	3	LSM6, RAD52
PHA-665752	1	EPHA4, FLJ25758, COX18, C12orf60
	2	SLC38A5, TNFRSF1B
	3	SEC63, TNFRSF14, CAPS, NECAP2, ROCK1
	4	—
PLX4720	1	SNCA, MRGPRX3
	2	ACP5
Paclitaxel	1	SLC35F5, ORC1, AQR, PTBP1
	2	BCL2L1
	3	LCP1
	4	—
Panobinostat	1	LARP6, MYOF

	2	KIRREL, EIF4EBP2, MIS18A, ATP9B, ZNF124, SLC2A1
	3	LOC100506779, DENND4B
	4	ZCCHC3, IL6, EIF4EBP2
RAF265	1	EMILIN2, CMTM3, ZNRF4, EXOSC9, RGS10, HMG20A, DUSP8, ZBTB41, IRS1, LOC100505986
Sorafenib	1	SLC9B2, DSTN, NCBP2, RAB30, TOMM22, ADAMTS15, PPME1, SNX21, CCDC28A, CWF19L1, EPHA4
	2	IKZF1, N4BP2L1
	3	BZRAP1
	4	NHSL1, LOC100507263, DCUN1D5, CCDC57, LIPG, NUPR1, NR2F6
TAE684	1	LAIR1, FLJ38717, GLCCI1, ELF
	2	LOC100507077, PPP4R1, S100A4, IGF2BP2, MCM3AP-AS1, LOC285286
TKI258	1	PKP3, HNRPDL, LAIR1, CXXC1
	2	LOC100130776, SLC39A13, RNF39, LAMA3, HMHA1
	3	—
Topotecan	1	SLFN11, ITPRIPL1, PRPF38A, THRB, HMGB2, HCLS1
	2	SLFN11, RPL4
	3	SMARCC1
	4	—
ZD-6474	1	MED23, NMI, CAPN1, MBTPS2, REL, PRKCQ, C17orf51, DYNLT3
