# Supplementary material for: Drug sensitivity prediction with high-dimensional mixture regression
Source: PLoS One. 2019 Feb 27;14(2):e0212108. doi: 10.1371/journal.pone.0212108 (PMC6392252; doi:10.1371/journal.pone.0212108)
Supplement: S5 Table — The values of corr(Ytrain,Y^train) and corr(Ytest,Y^test) produced by support vector regression ({linear, radial, polynomial, sigmoid} ×{eps-regression, nu-regression}), random forest, ridge regression and elastic net for the CCLE dataset. (PDF) [file pone.0212108.s005.pdf]

Table S5

Drug	linear & eps-reg		linear & nu-reg		radial & eps-reg		radial& nu-reg		poly& eps-reg		poly &nu-reg		sigmoid &eps-reg		sigmoid & nu-reg		random forest		ridge		elastic net	
	Fit	Pred	Fit	Pred	Fit	Pred	Fit	Pred	Fit	Pred	Fit	Pred	Fit	Pred	Fit	Pred	Fit	Pred	Fit	Pred	Fit	Pred
17-AAG	0.995	0.416	1	0.332	0.950	0.569	0.948	0.571	0.923	0.502	0.926	0.504	0.704	0.546	0.710	0.571	0.589	0.543	0.789	0.589	0.776	0.518
AEW541	0.995	0.138	1	0.061	0.934	0.400	0.936	0.405	0.942	0.439	0.942	0.438	0.576	0.358	0.640	0.465	0.530	0.390	0.743	0.444	0.707	0.339
AZD0530	0.995	0.197	1	0.148	0.881	0.385	0.885	0.382	0.921	0.381	0.925	0.381	0.563	0.374	0.579	0.349	0.392	0.364	0.701	0.357	0.637	0.336
AZD6244	0.995	0.427	1	0.336	0.939	0.702	0.939	0.703	0.937	0.633	0.937	0.633	0.699	0.585	0.737	0.647	0.642	0.691	0.772	0.682	0.775	0.691
Erlotinib	0.995	0.189	1	0.151	0.856	0.471	0.860	0.471	0.864	0.436	0.866	0.438	0.469	0.375	0.542	0.421	0.448	0.433	0.624	0.427	0.583	0.370
Irinotecan	0.996	0.593	1	0.556	0.943	0.639	0.940	0.639	0.891	0.623	0.885	0.621	-0.040	-0.085	0.096	0.041	0.711	0.684	0.785	0.645	0.826	0.701
L-685458	0.995	0.280	1	0.236	0.871	0.505	0.873	0.505	0.904	0.490	0.904	0.491	0.330	0.465	0.003	-0.255	0.539	0.604	0.680	0.581	0.646	0.532
LBW242	0.995	0.134	1	0.112	0.829	0.156	0.822	0.158	0.793	0.112	0.789	0.105	0.443	0.144	0.521	0.165	0.149	0.151	0.798	0.268	0.626	0.265
Lapatinib	0.996	0.531	1	0.504	0.885	0.451	0.887	0.454	0.945	0.544	0.945	0.544	0.378	0.306	0.548	0.417	0.563	0.524	0.668	0.473	0.687	0.518
Nilotinib	0.995	0.077	1	0.023	0.837	0.372	0.830	0.358	0.956	0.317	0.957	0.299	-0.032	-0.145	0.023	-0.074	0.484	0.440	0.699	0.428	0.755	0.338
Nutlin-3	0.995	0.095	1	0.068	0.886	0.324	0.891	0.316	0.917	0.382	0.918	0.379	0.485	0.214	0.496	0.225	0.389	0.362	0.608	0.303	0.585	0.234
PD-0325901	0.995	0.572	1	0.480	0.950	0.773	0.950	0.773	0.953	0.714	0.953	0.716	0.738	0.700	0.757	0.713	0.684	0.753	0.800	0.731	0.806	0.731
PD-0332991	0.996	0.156	1	0.126	0.882	0.469	0.882	0.465	0.863	0.422	0.860	0.419	0.293	0.368	0.283	0.386	0.558	0.543	0.652	0.527	0.635	0.504
PF2341066	0.995	0.157	1	0.118	0.844	0.393	0.844	0.395	0.827	0.300	0.827	0.303	0.208	0.067	0.159	0.029	0.547	0.467	0.669	0.439	0.678	0.393
PHA-665752	0.995	-0.029	1	-0.028	0.898	0.269	0.901	0.271	0.863	0.324	0.863	0.319	0.542	0.144	0.571	0.116	0.463	0.314	0.703	0.193	0.652	0.204
PLX4720	0.995	0.416	1	0.346	0.889	0.610	0.890	0.610	0.899	0.501	0.898	0.501	0.606	0.534	0.622	0.552	0.513	0.481	0.725	0.551	0.703	0.508
Paclitaxel	0.996	0.237	1	0.183	0.922	0.668	0.922	0.659	0.883	0.610	0.879	0.596	-0.021	-0.019	0.282	0.239	0.557	0.604	0.729	0.652	0.810	0.574
Panobinostat	0.995	0.325	1	0.258	0.941	0.577	0.937	0.587	0.919	0.531	0.913	0.538	0.364	0.290	0.379	0.305	0.677	0.598	0.750	0.591	0.750	0.591
RAF265	0.996	0.400	1	0.376	0.920	0.585	0.923	0.598	0.916	0.511	0.915	0.510	0.587	0.442	0.631	0.465	0.513	0.521	0.735	0.538	0.720	0.481
Sorafenib	0.995	0.217	1	0.149	0.872	0.411	0.874	0.413	0.942	0.452	0.940	0.451	0.305	0.153	0.320	0.149	0.495	0.362	0.654	0.404	0.610	0.348
TAE684	0.995	0.182	1	0.141	0.899	0.462	0.901	0.460	0.898	0.404	0.893	0.400	0.146	0.029	0.125	-0.012	0.497	0.429	0.689	0.438	0.712	0.446
TKI258	0.995	0.162	1	0.123	0.889	0.349	0.890	0.352	0.912	0.434	0.912	0.429	0.114	-0.050	0.280	0.289	0.472	0.310	0.668	0.335	0.711	0.306
Topotecan	0.995	0.381	1	0.341	0.931	0.615	0.926	0.618	0.884	0.601	0.873	0.603	0.327	0.387	0.312	0.359	0.662	0.648	0.725	0.627	0.761	0.647
ZD-6474	0.996	0.232	1	0.206	0.921	0.470	0.923	0.468	0.903	0.450	0.904	0.447	0.610	0.393	0.642	0.377	0.465	0.484	0.753	0.432	0.689	0.344
